# Comprehensive Analysis of Cell Population Dynamics and Related Core Genes During Vitiligo Development

**DOI:** 10.3389/fgene.2021.627092

**Published:** 2021-02-19

**Authors:** Jingzhan Zhang, Shirong Yu, Wen Hu, Man Wang, Dilinuer Abudoureyimu, Dong Luo, Tingting Li, Linglong Long, Hui Zeng, Chao Cheng, Zixian Lei, Jianan Teng, Xiaojing Kang

**Affiliations:** ^1^Department of Dermatology, People’s Hospital of Xinjiang Uygur Autonomous Region, Urumqi, China; ^2^Xinjiang Key Laboratory of Dermatology Research, Urumqi, China; ^3^Department of Gastroenterology, People’s Hospital of Xinjiang Uygur Autonomous Region, Urumqi, China; ^4^Center for Genome Analysis, ABLife Inc., Wuhan, China; ^5^Medical School, Shihezi University, Shihezi, China

**Keywords:** vitiligo, bioinformatics, differentially expressed genes, immune infiltration, coexpression analysis

## Abstract

Vitiligo is a common immune-related depigmentation condition, and its pathogenesis remains unclear. This study used a combination of bioinformatics methods and expression analysis techniques to explore the relationship between immune cell infiltration and gene expression in vitiligo. Previously reported gene expression microarray data from the skin (GSE53146 and GSE75819) and peripheral blood (GSE80009 and GSE90880) of vitiligo patients and healthy controls was used in the analysis. R software was used to filter the differentially expressed genes (DEGs) in each dataset, and the KOBAS 2.0 server was used to perform functional enrichment analysis. Compared with healthy controls, the upregulated genes in skin lesions and peripheral blood leukocytes of vitiligo patents were highly enriched in immune response pathways and inflammatory response signaling pathways. Immunedeconv software and the EPIC method were used to analyze the expression levels of marker genes to obtain the immune cell population in the samples. In the lesional skin of vitiligo patients, the proportions of macrophages, B cells and NK cells were increased compared with healthy controls. In the peripheral blood of vitiligo patients, CD8+ T cells and macrophages were significantly increased. A coexpression analysis of the cell populations and DEGs showed that differentially expressed immune and inflammation response genes had a strong positive correlation with macrophages. The TLR4 receptor pathway, interferon gamma-mediated signaling pathway and lipopolysaccharide-related pathway were positively correlated with CD4+ T cells. Regarding immune response-related genes, the overexpression of *IFITM2, TNFSF10, GZMA, ADAMDEC1, NCF2, ADAR, SIGLEC16*, and *WIPF2* were related to macrophage abundance, while the overexpression of *ICOS, GPR183, RGS1, ILF2* and *CD28* were related to CD4+ T cell abundance. *GZMA* and *CXCL10* expression were associated with CD8+ T cell abundance. Regarding inflammatory response-related genes, the overexpression of *CEBPB, ADAM8, CXCR3*, and *TNIP3* promoted macrophage infiltration. Only *ADORA1* expression was associated with CD4+ T cell infiltration. *ADAM8* and *CXCL10* expression were associated with CD8+ T cell abundance. The overexpression of *CCL18, CXCL10, FOS, NLRC4, LY96, HCK, MYD88*, and *KLRG1*, which are related to inflammation and immune responses, were associated with macrophage abundance. We also found that immune cells infiltration in vitiligo was associated with antigen presentation-related genes expression. The genes and pathways identified in this study may point to new directions for vitiligo treatment.

## Introduction

Vitiligo is a common depigmentation condition caused by the destruction of epidermal melanocytes (Ezzedine et al., 2015). It can occur at any age, but onset is most common in childhood or adolescence. According to the clinical characteristics of the skin lesions, it is mainly divided into segmental vitiligo and non-segmental vitiligo. The vast majority of patients have non-segmental vitiligo. Vitiligo has a long course and poor curative effect, placing a large economic and psychological burden on patients. Vitiligo patients have a higher frequency of autoimmune disorders compared to the general population; such disorders include thyroid disease, rheumatoid arthritis, multiple sclerosis, lupus erythematosus, and alopecia areata (Alikhan et al., 2011; Hadi et al., 2020). The etiology and pathogenesis of vitiligo are not fully understood. It is currently believed to be mainly related to genetic, immune, oxidative stress, neuromodulation, and other factors (Richmond et al., 2013; Iannella et al., 2016; Rodrigues et al., 2017).

In recent years, the research on the pathogenesis of vitiligo has mainly focused on the intrinsic damage of melanocytes and the specific immune response mediated by T cells. Cytotoxic T lymphocytes and a variety of cytokines, including the CXC family included CXCR3 and CXCR6, as well as the chemokines CXCL9, CXCL10, CXCL11, and CXCL16(the only known natural ligand of CXCR6), play an important role in the pathogenesis of vitiligo (van den Boorn et al., 2009; Rashighi et al., 2014; Li et al., 2017; Aguilera-Durán and Romo-Mancillas, 2020). There were higher percentages of CXCR3(+) CD8(+) T cells in vitiligo patients compared with controls, while the expression of CXCR3(+) CD4(+) T cells also increased in patients with progressive vitiligo (Wang X. X. et al., 2016). In addition to CD8+ T cell infiltration at the edge of vitiligo skin lesions, innate immune cells such as natural killer (NK) cells, inflammatory dendritic cells (DCs), and macrophages are also observed, indicating that the innate immune response is involved in the occurrence and development of vitiligo, Except for CD8+ T cells, the role of these cells in the pathogenesis of vitiligo is still unclear. Oxidative stress as an initiating factor can participate in the initiation of specific T cell immune responses against melanocytes by activating the innate immune response, causing melanocyte damage (Wang et al., 2019). Approximately 9% of patients with vitiligo have a family history of the condition, and its inheritance involves multiple genes, including the autoimmune susceptibility-related genes *SLEV1, DNMT1, IL-10, TGFBR2, UVRAG*, and *MYG1* (Mohammed et al., 2015). Genome-wide association studies have identified 50 contributory loci associated with vitiligo (Quan et al., 2010; Shen et al., 2016; Jin et al., 2019; Roberts et al., 2019). In the occurrence and development of vitiligo, the immune cell infiltration and the abnormal expression of specific genes are closely related to the pathogenesis. Differences in gene expression may be related to the types of immune cells.

With the emergence and development of high-throughput research methods, high-throughput data analysis and information screening have become important methods for studying disease pathogenesis. In recent years, some vitiligo-related bioinformatics studies have been published (Rashighi et al., 2014; Wang P. et al., 2016; Dey-Rao and Sinha, 2017; Singh et al., 2017). However, these studies were based on a single cohort study, with poor reproducibility and consistency. These studies did not consider changes in the levels of multiple immune cells and their association with differentially expressed genes (DEGs). To overcome these limitations, the current study combined comprehensive bioinformatics methods with expression analysis techniques to explore the pathogenesis of vitiligo based on the previously reported gene expression data of skin samples (GSE53146 and GSE75819) and peripheral blood samples (GSE80009 and GSE90880) of vitiligo patients and healthy controls. We first identified DEGs in each dataset and then performed DEG functional annotation and pathway analysis. We also analyzed the immune cell populations of the different datasets. A coexpression analysis of the cell populations and DEGs was performed to investigate the gene expression specificity of immune cells in vitiligo skin and peripheral blood. The findings provide insight into the specific mechanisms of immune and inflammatory responses in vitiligo and can be used to identify new therapeutic targets.

## Materials and Methods

### Data Introduction and Data Preprocessing

**FIGURE 1:**
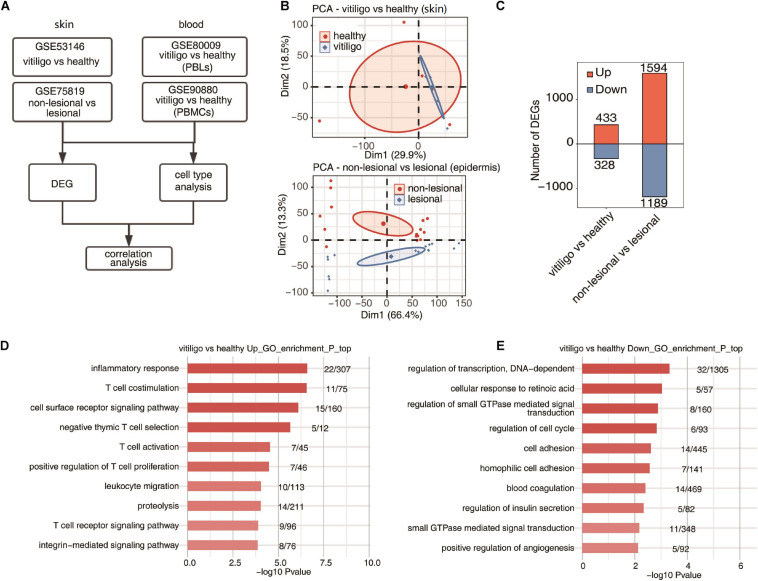
Specificity of upregulated expression of immune response gene of skin between vitiligo patients and healthy controls. **(A)** Workflow of this study. **(B)** Principal component analysis (PCA) of vitiligo vs healthy samples (up) and non-lesional vs lesional dataset (down) based on normalized gene expression level. The samples were grouped by disease state and the ellipse for each group is the confidence ellipse. **(C)** The number of DEGs. The number of upregulated and downregulated DEGs were showed in bar plot. **(D,E)** Top 10 most enriched GO terms of upregulated **(D)** and downregulated **(E)** genes from vitiligo vs healthy samples.

The workflow of this study is shown in Figure 1A. We used “vitiligo” as a keyword to retrieve and select the dataset that met the requirements. The Gene Expression Omnibus database (GEO)^1^ was searched. The data from skin and peripheral blood samples were collected. Four gene expression profiles (GSE75819, GSE53146, GSE90880, and GSE80009) were identified for use in our comprehensive analysis; all datasets were expression chip data. The expression profiles were converted and standardized by log2 to obtain a series matrix file. The microarray data of GSE75819 were based on the GPL6884 platform (Illumina HumanWG-6 v3.0 expression beadchip) and included lesional and non-lesional epidermis samples from 15 patients with non-segmental vitiligo. The microarray data of GSE53146 were based on the GPL14951 platform (Illumina HumanHT-12 WG-DASL V4.0 R2 expression beadchip) and included 10 skin samples—5 from vitiligo skin lesions and 5 from healthy controls. The microarray data of GSE90880 were based on GPL8300 (Affymetrix Human Genome U95 version 2 array). RNA was extracted from separated peripheral blood mononuclear cells (PBMCs); six samples were from non-segmental vitiligo patients and eight were from healthy controls. The microarray data of GSE80009 were based on GPL16951 (Phalanx Human OneArray ver. 6 release 1). RNA was extracted from separated peripheral blood leukocytes (PBLs); four samples were from non-segmental vitiligo patients and four were from healthy controls.

### Identification of DEGs

The raw data of the included datasets were used for integrated analysis using the affy package of R (Gautier et al., 2004). The LIMMA package was used to identify DEGs (Law et al., 2016). *P*-values <0.05 and | log2-fold change (FC) | ≥1.5 or ≤2/3 were considered as threshold values for DEG identification. The Ggplot2 package and the pheatmap package in R were used to draw volcano plots and heat maps, respectively (Wang et al., 2014).

### PCA of DEGs and Correlation Analyses

Principal component analysis (PCA) is a multiple regression analysis and was used to assess the DEGs. We used PCA analysis to examine the overall gene expression patterns. The differential expression between the case group and the control group was observed using DEGs as variable. PCA analysis was performed on each expression dataset. The factoextra package in R was used for data processing, analysis and mapping.

### Functional Enrichment Analysis

Gene Ontology (GO) terms and Kyoto Encyclopedia of Genes and Genomes (KEGG) pathways were identified using the KOBAS 2.0 server to investigate the comprehensive set of functional annotations of a large list of genes. The Benjamini-Hochberg FDR controlling procedure and the hypergeometric test were used to define the enrichment of each term. Reactome^2^ pathway profiling was also used for the functional enrichment analysis of the sets of selected genes. A *p*-value <0.005 was considered as the cutoff criterion.

### Analysis of Immune Cell Populations

The expression levels of marker genes were analyzed to obtain the immune cell populations in the samples. The software used for cell component analysis was immunedeconv,^3^ which provides an integrated environment to manage different cell component analysis software. The results were obtained using the EPIC method^4^ since a comparison with other methods demonstrated improved accuracy for EPIC. For each dataset, gene expression profiles were used as input, and analyzed with default parameters. The output cell population results were used for comparison and visualization (Racle et al., 2017).

### Coexpression Analysis of Cell Populations and DEGs

Using the previously reported data, a coexpression analysis of the cell population and DEGs in the peripheral blood and skin of vitiligo patients and healthy controls was carried out according to similar study (Li et al., 2016). We calculated Pearson correlation coefficients between the cell infiltration and immune response-related or inflammatory response-related gene expression in three datasets.

## Results

### Identification of DEGs, PCA, and Function Analysis in Tissue Samples of Vitiligo Patients

The venn diagrams in Supplementary Figure 1 showed the shared probes and DEGs identified from the four transcription profile datasets (GSE53146, GSE75819, GSE80009, and GSE90880). In the skin specimens, the gene expression profiling of GSE53146 contained 10 skin samples including epidermis and superficial dermis, 5 from vitiligo patients and 5 from healthy controls. The gene expression profiling of GSE75819 included lesional and non-lesional epidermis samples of 15 patients. The PCA of vitiligo vs control samples and non-lesional vs lesional datasets based on normalized gene expression levels are shown in Figure 1B. The sample set was not completely continuous. The volcano plot of DEGs is shown in Supplementary Figure 2A. A total of 761 DEGs in GSE53146 were identified, including 433 upregulated genes and 328 downregulated genes; 2,783 DEGs were identified in GSE75819, including 1,594 upregulated genes and 1,189 downregulated genes (Figure 1C).

In the gene expression profiling of GSE53146, the GO term enrichment analysis showed that the upregulated DEGs were significantly enriched in “inflammatory response, T cell costimulation, cell surface receptor signaling pathway, T cell activation, negative thymic T cell selection, positive regulation of T cell proliferation, leukocyte migration, proteolysis, T cell receptor signaling pathway, and integrin-mediated signaling pathway” (Figure 1D). The downregulated DEGs were significantly enriched in “regulation of transcription, cellular response to retinoic acid, DNA-dependent, regulation of small GTPase mediated signal transduction, regulation of cell cycle, cell adhesion, homophilic cell adhesion, blood coagulation, regulation of insulin secretion, small GTPase mediated signal transduction and positive regulation of angiogenesis” (Figure 1E). The KEGG pathway analysis was further performed, and the top five pathways enriched in upregulated DEGs were “primary immunodeficiency, natural killer cell mediated cytotoxicity, cell adhesion molecules (CAMs), T cell receptor signaling pathway, and cytokine–cytokine receptor interaction,” while “melanogenesis, galactose metabolism, regulation of autophagy, tyrosine metabolism, cholinergic synapse, folate biosynthesis, and pathways in cancer” were enriched in downregulated DEGs.

In the gene expression profiling of GSE75819, the GO analysis showed that the upregulated DEGs were mainly enriched in “translation, gene expression, mitotic cell cycle, RNA metabolic process, viral reproduction, nonsense–mediated decay, mRNA metabolic process, nuclear-transcribed mRNA catabolic process, cellular metabolic process, SRP-dependent co-translational protein targeting to membrane and respiratory electron transport chain” (Supplementary Figure 2B). The downregulated DEGs were mainly enriched in “small molecule metabolic process, carbohydrate metabolic process, *in utero* embryonic development, membrane organization, cell death, axon guidance, Notch signaling pathway, glycosaminoglycan metabolic process, skeletal muscle cell differentiation and glucose metabolic process” (Supplementary Figure 2C). The KEGG pathway analysis found that the top ten pathways enriched in upregulated DEGs were “ribosome, oxidative phosphorylation, proteasome, basal transcription factors, Parkinson’s disease, Huntington’s disease, citrate cycle (TCA cycle), p53 signaling pathway, carbon metabolism and cell cycle,” while “acute myeloid leukemia, chronic myeloid leukemia, melanogenesis, endocytosis, ErbB signaling pathway, amino sugar and nucleotide sugar metabolism, fructose and mannose metabolism, HIF–1 signaling pathway, glycosaminoglycan biosynthesis-heparan sulfate/heparin and riboflavin metabolism” were mainly enriched by downregulated DEGs. The above results show that the expression of T cell activation and inflammatory response genes was selectively upregulated in the skin of vitiligo patients compared to healthy controls, but there was no differential expression of these genes between the lesion and non-lesion epidermis samples of vitiligo patients.

**FIGURE 2:**
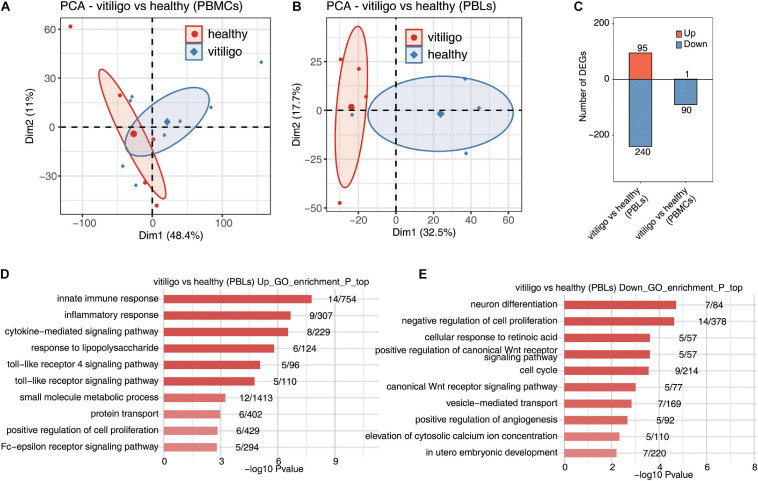
Deregulated gene expression of vitiligo patients compared to healthy controls in peripheral blood mononuclear cells and leukocytes. **(A,B)** Principal component analysis (PCA) of vitiligo and healthy samples from vitiligo vs healthy (PBLs) dataset **(A)** and vitiligo vs healthy (PBMCs) dataset **(B)** based on normalized gene expression level. The samples were grouped by disease state and the ellipse for each group is the confidence ellipse. **(C)** The number of DEGs. The number of upregulated and downregulated DEGs were showed in bar plot. **(D,E)** Top 10 most enriched GO terms of upregulated **(D)** and downregulated **(E)** genes from vitiligo vs healthy samples (PBLs).

In the peripheral blood specimens, the gene expression profiling of GSE80009 included eight peripheral blood samples, four from vitiligo patients and four from healthy controls. The gene expression profiling of GSE90880 included 14 peripheral blood samples, six from vitiligo patients and eight from healthy controls. The PCA of vitiligo vs healthy control peripheral blood sample data based on normalized gene expression levels is shown in Figures 2A,B. There was a clear spatial separation of the samples. The volcano plot of DEGs is shown in Supplementary Figure 2A. A total of 335 DEGs in GSE80009 were identified in vitiligo peripheral blood samples when compared with the expression profiles of controls, including 95 upregulated genes and 240 downregulated genes. A total of 91 DEGs in GSE90880 were identified in vitiligo samples compared with the expression profiles of controls, including 1 upregulated gene and 90 downregulated genes (Figure 2C).

In the gene expression profiling of GSE80009, the GO term enrichment analysis showed that the upregulated DEGs were significantly enriched in “inflammatory response, innate immune response, cytokine–mediated signaling pathway, small molecule metabolic process, response to lipopolysaccharide, toll-like receptor (TLR) signaling pathway, Fc–epsilon receptor signaling pathway, TLR 4 signaling pathway, protein transport and positive regulation of cell proliferation” (Figure 2D). The downregulated DEGs were mainly enriched in “neuron differentiation, negative regulation of cell proliferation, cellular response to retinoic acid, positive regulation of canonical Wnt receptor signaling pathway, cell cycle, canonical Wnt receptor signaling pathway, vesicle–mediated transport, positive regulation of angiogenesis, elevation of cytosolic calcium ion concentration and *in utero* embryonic development” (Figure 2E). The KEGG pathway analysis showed that the upregulated DEGs were enriched in “Toll-like receptor signaling pathway,” while no signaling pathways were enriched in downregulated DEGs.

In the gene expression profiling of GSE90880, the GO analysis showed that the downregulated DEGs were significantly enriched in “type I interferon-mediated signaling pathway, cytokine-mediated signaling pathway, defense response to virus, innate immune response, response to virus, negative regulation of viral genome replication, defense response, antigen processing and presentation of exogenous peptide antigen via MHC class I,TAP–dependent, antigen processing and presentation of exogenous peptide antigen and peptide antigen via MHC class I.” KEGG pathway analysis was further performed, “measles, influenza A, sphingolipid metabolism, herpes simplex infection, proteasome, TLR signaling pathway and systemic lupus erythematosu.” The KEGG pathway analysis revealed that “measles, influenza A, sphingolipid metabolism, herpes simplex infection, proteasome, TLR signaling pathway, systemic lupus erythematosus, adherens junction, cytosolic DNA-sensing pathway and RIG-I-like receptor signaling pathway” were enriched in downregulated DEGs (Supplementary Figure 3). No GO or KEGG pathways were enriched in upregulated DEGs. The downregulated genes in PBLs from vitiligo patients were highly enriched in the innate immune response and inflammatory response pathways; these findings were similar to those of the vitiligo skin samples. No similar finding were identified for the monocytes.

### Analysis of Immune Cell Populations

**FIGURE 3:**
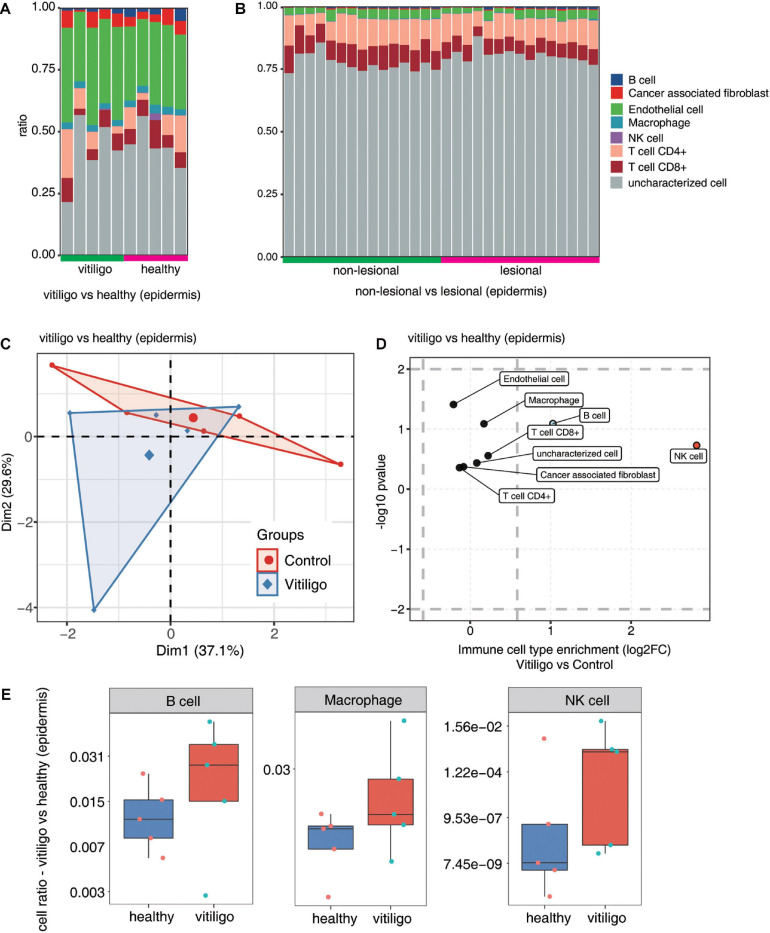
Increase of macrophage, B cells and NK cells population in epidermis of vitiligo patients compared to healthy controls. **(A,B)** Fractions of cell type estimated by EPIC in each sample. **(C)** Principal component analysis (PCA) of samples based on proportion of different cell types. The samples were grouped by disease state and the ellipse for each group is the confidence ellipse. **(D)** Scatter plot for the enrichment of each cell type in vitiligo patients compared to healthy controls. *X*-axis: log fold change of mean cell fraction of advanced DN compared healthy controls. *Y*-axis: log *p* value using Students *t*-test. **(E)** Box plots showing proportion of each cell type in vitiligo and healthy samples.

We analyzed differences in the immune cell populations in skin samples of vitiligo patients vs healthy controls, as well as differences in immune cell populations of skin lesion vs non-lesion epidermis samples from vitiligo patients. The fractions of the various cell types were estimated by EPIC, and the data were analyzed by PCA. Scatter plots showed the cell type enrichments, and box plots showed the proportion of each cell type (Figure 3 and Supplementary Figure 4). The PCA showed a spatial separation of samples without outliers or batch effects. The macrophage, B cell and NK cell populations were increased in the skin of vitiligo patients compared to healthy controls. No such differences were found between the lesion and non-lesion samples of vitiligo patients.

**FIGURE 4:**
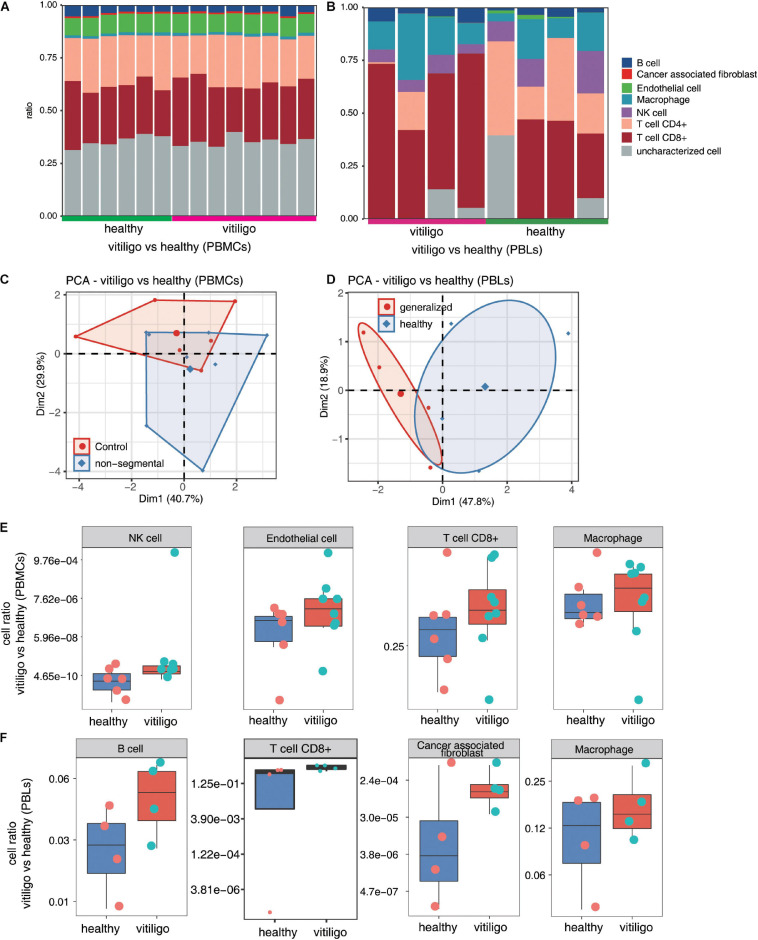
Increase of CD8+ T cell and macrophage population in peripheral blood of vitiligo patients compared to healthy controls. **(A,B)** Fractions of cell type estimated by EPIC in each sample. **(C,D)** Principal component analysis (PCA) of samples based on proportion of different cell types. The samples were grouped by disease state and the ellipse for each group is the confidence ellipse. **(E,F)** Box plots showing proportion of each cell type from vitiligo vs healthy (PBMCs) dataset **(E)** and vitiligo vs healthy (PBLs) dataset **(F)**.

The immune cell populations in peripheral blood of vitiligo patients and healthy controls were also compared (Figure 4 and Supplementary Figure 5). The CD8+ T cell and macrophage populations were increased in the peripheral blood of non-segmental vitiligo patients compared to controls. The overall CD8+ T cell and macrophage populations were higher in PBLs than in PBMCs.

### Coexpression Analysis of Cell Populations and DEGs

**FIGURE 5:**
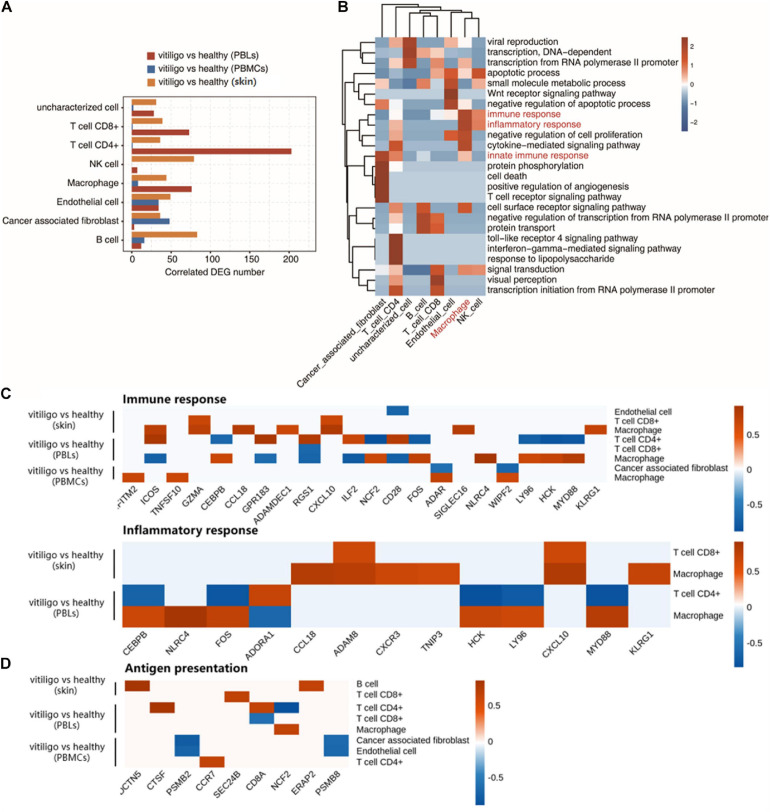
Coexpression analysis of cell population and differential expressed genes in peripheral blood and skin of vitiligo patients. **(A)** Bar plot shows cell type ratio and the number of coexpressed DEGs in three datasets. **(B)** Top 10 most enriched GO terms (biological process) by cell type coexpressed DEGs in three datasets. **(C)** Association of cell infiltration and immune response related (up) or inflammatory response related gene expression in three datasets (down). **(D)** Association of antigen presentation related gene expression in three datasets.

Through the above analysis, significant differences in the activation of epidermal T cells and the expression of inflammatory response genes were found between the vitiligo and healthy control groups, but there were no similar differences between the vitiligo lesion and non-lesion groups. Therefore, we conducted a coexpression analysis of DEGs and the cell populations of vitiligo patients and healthy controls. The cell type populations and the coexpressed DEGs in three datasets are shown in Figure 5A and Supplementary Figure 6. The correlation analysis between cell population and DEGs showed that the expression of inflammation and immune response genes had a strong positive correlation with macrophages. Innate immunity and T cell receptor signal transduction were highly correlated with tumor-related fibroblasts. The TLR4 receptor pathway and interferon gamma-mediated signaling pathway were highly positively correlated with CD4+ T cells (Figure 5B). We drew correlation diagrams between cell infiltration and immune response- or inflammatory response-related genes expression in the three datasets. For immune response-related genes, we observed positive correlations between *IFITM2, ICOS, TNFSF10, GZMA, CCL18, ADAMDEC1, CXCL10, NCF2, FOS, ADAR, SIGLEC16, NLRC4, WIPF2, LY96, HCK, MYD88*, and *KLRG1* expression and macrophage abundance. *ICOS, GPR183, RGS1, ILF2*, and *CD28* expression were associated with CD4+ T cell abundance. *GZMA* and *CXCL10* expression were associated with CD8+ T cell abundance. For inflammatory response-related genes, *CEBPB, NLRC4, FOS, CCL18, ADAM8, CXCR3, TNIP3, HCK, LY96, CXCL10, MYD88*, and *KLRG1* expression were positively correlated with macrophage abundance. *ADAM8* and *CXCL10* expression were associated with CD8 + T cell abundance. Only *ADORA1* expression was associated with CD4+ T cells infiltration (Figure 5C). We also drew correlation diagrams between cell infiltration and antigen presentation-related genes expression in the three datasets. We observed positive correlations between *DCTN5* and *ERAP2* expression and B cell abundance. *CTSF, CDBA*, and *CCR7* expression were associated with CD4 + T cell abundance. *SEC24B* expression were associated with CD8+ T cell abundance. *NCF2* expression were associated with macrophage abundance (Figure 5D). The expression of adhesion molecules in endothelial cells in the two skin datasets were analyzed. No adhesion molecules-related genes were found in GSE53146, and the negative correlation genes *CD28, MPZL1* were found in GSE75819.

## Discussion

The development of vitiligo is accompanied by the activation and infiltration of immune cells in peripheral blood and the skin. Many studies have reported the role of T cell activation in vitiligo; however, few studies have investigated the abnormal regulation of other immune cells in this condition, such as macrophages, B cells, NK cells and endothelial cells. Using previously reported gene expression data from skin and peripheral blood samples of vitiligo patients and healthy controls, we applied bioinformatics methods to identify the DEGs in each dataset. We then performed functional annotation and pathway analysis of the DEGs. We also analyzed differences in immune cell populations. Subsequently, a coexpression analysis of the cell populations and DEGs was performed to study the characteristics of specific immune cell infiltration in the skin and peripheral blood of patients with vitiligo and their association with DEGs. Considering the high variability between published datasets, we also screened overlapping genes in the datasets of skin and blood samples respectively. The names from the first gene expression dataset were queried in the second dataset in Excel using ISERROR function and VLOOKUP function. Values that were retrieved as FALSE were then queried for alternate gene names using GeneCards.org. Missing values were renamed to match the naming conventions used across all datasets, which yielded overlapping genes across the datasets. These genes were then compared for both p value and directionality across datasets, and conserved gene expression patterns were presented in the results. The two skin datasets shared 13,069 genes, and the two blood datasets shared 2,000 genes. We used the expression levels of the two combined blood datasets to do a heatmap test. The test indicated that the expression levels of different datasets were very heterogeneous and cannot be directly compared. Therefore, we first performed a difference analysis within the same dataset, and then compared the DEGs between different datasets.

The upregulated genes in the skin of vitiligo patients and healthy controls are highly enriched in T cell activation, T cell costimulation, T cell receptor signaling, the positive regulation of T cell proliferation, negative thymic T cell selection and other T cell-related immune pathways. These findings are consistent with previous studies (van den Boorn et al., 2009; Cheuk et al., 2017; Li et al., 2017; Boniface et al., 2018; Molodtsov and Turk, 2018; Riding and Harris, 2019; Willemsen et al., 2019; Aghamajidi et al., 2020). The results also revealed the response of new T cell activity in the pathogenesis of vitiligo. The upregulated genes also highly enriched the inflammatory response and cell surface receptor signaling pathways. These findings increase our understanding of the regulatory network of gene expression in vitiligo. By contrast, there were no differences in immune-related genes between the lesion and non-lesion epidermis samples of vitiligo patients. The upregulated genes in PBLs of vitiligo patients compared with healthy controls were highly enriched in the innate immune response and inflammatory response pathways; a similar enrichment trend was found between the skin samples of vitiligo patients and healthy controls. No similar enrichment trends in PBMCs were found when comparing vitiligo patients and healthy controls. We speculate that the activation of T cells and the high expression of inflammatory response genes in the skin are closely related to the activity of immune cells in the peripheral blood of these patients.

We analyzed the immune cell populations in the skin and peripheral blood chip data. The proportion of innate immune cells, such as NK cells, macrophages, and inflammatory DCs, in skin samples of vitiligo patients were higher than in healthy controls. This suggested that the innate immune response is involved in the occurrence and progression of vitiligo. Damage-associated molecular patterns (DAMPs) are an important part of innate immunity (Roh and Sohn, 2018). The heat shock protein HSP70 is the most prominent molecule among the many DAMP molecules of vitiligo. One study found that under the stress of the phenolic agent 4-TBP, HSP70 promotes the activation and migration of DCs by binding to the CD91 receptor on the surface of DCs (Mosenson et al., 2012). Transcriptome data was used to investigate the proportion of immune cell types in PML and PBMC samples from vitiligo patients compared with healthy controls. There were larger populations of CD8+ T cells and macrophages in the non-segmental vitiligo patient peripheral blood samples compared to controls. The overall CD8+ T cell and macrophage populations in PBLs were higher than in PBMCs. This is consistent with the enrichment results of the above immune pathways. One study found that the peripheral blood T-helper type 17 reaction was unbalanced in patients with vitiligo, and the plasma levels of IL-17A and IL-22 were higher healthy controls, which was consistent with our results (Behfarjam et al., 2018).

The correlation analysis between cell population changes and DEGs showed that there was a strong positive correlation between the differential expression of inflammatory and immune response genes and macrophages. Previous studies have found that macrophage inhibitory factor (*MIF*) gene polymorphisms and serum MIF and MIF mRNA levels are significantly higher in vitiligo patients than in healthy people, which was consistent with our results (Oiso et al., 2013; Farag et al., 2018; Garcia-Orozco et al., 2020). Innate immunity and T cell receptor signal transduction are highly correlated with human cancer-associated fibroblasts (CAFs). CAFs, macrophage type 2 cells and regulatory T cells (Tregs) may produce an immune barrier against the anti-tumor immune response mediated by CD8+ T lymphocytes (Farhood et al., 2019). TLRs play a role in the pathogenesis of vitiligo (Traks et al., 2015). The TLR4 receptor pathway is highly positively correlated with CD4+ T cells. CD4+ T lymphocytes express functional TLR4, and the TLR4-induced response is mediated by *MIF*. *MIF* can prevent excessive TCR/CD3-mediated activation of T lymphocytes. *MIF* can also enable activated CD4+ T lymphocytes perceive their microenvironment and modulate their effector response through TLR4 (Alibashe-Ahmed et al., 2019; Ostareck and Ostareck-Lederer, 2019). The anti-inflammatory response of CD4+ T regulatory cells is modulated by tumor necrosis factor-alpha (TNFα)- and TLR4-dependent pathways in the murine burn injury model (Bock et al., 2018).

The results of this study showed that the levels of macrophages, B cells, NK cells, and T cells increased in the development of vitiligo. The levels of CD8+ T cells and macrophages were increased in the peripheral blood of vitiligo patients. Macrophage can clear the melanocyte-derived deposits, which is essential for the recoloration of vitiligo (Oiso et al., 2013). Inflammatory cells including macrophages, T cytotoxic and helper cells regulate immune activities by antigen presentation to T cells or cytokine production in vitiligo (Srivastava et al., 2021). B cells and germinal center reactions are involved in the pathogenesis of vitiligo (Raam et al., 2018). Self-reactive B cells activated by B lymphocyte activating factor may function as cellular adjuvants to activate CD4+ T cells, enhancing their auxiliary effect on CD8+ T cell activation. B cells activated by B lymphocyte activating factor capture antigen directly presented to CD8+ T cells. B lymphocyte activating factor transmits a complete costimulatory signal to T cells, and plays an additional role in the autoimmune response of vitiligo. Activated B cells capture antigens and present them directly to CD8+ T cells. In addition, B lymphocyte activating factor can transmit costimulatory signals to T cells, and plays an additional role in the autoimmune response of vitiligo (Lin et al., 2011). NK cells are the first line of defense against early cell transformation, viral infections, microbial infections and tumor growth, and are part of the group 1 innate lymphoid cells (Sonnenberg and Artis, 2015). NK cells can directly regulate the expression of interferon gamma (IFNγ), melanocytes activated by IFNγ express co-stimulatory factors, trigger T cell proliferation and anti-melanocyte immunity (Tulic et al., 2019). One study reported the NK cells dependent immune response induced by monobenzone, which was completely intact in the absence of T and B cells in a setting of contact hypersensitivity (van den Boorn et al., 2016). Time series analysis is needed to demonstrate whether increased T cells lead to the recruitment or activation of other cell types.

There is a relationship between DEGs and the immune cell types in the development of vitiligo. The infiltration of macrophages and CD4+ T lymphocytes was found to be associated with the expression of immune response-related or inflammatory response-related genes. At present, only a few of these genes and their expression products have been confirmed in the pathogenesis of vitiligo. For example, CXCL9 and CXCL10 induced by IFN-γ play a key role in the skin migration of CD8+ T cells in vitiligo, and the expression of *CXCR3* on melanocyte-specific CD8+ T cells has been detected in skin lesions and in the peripheral blood of vitiligo patients (Rashighi et al., 2014). Additionally, a clinical study confirmed that CXCL9 and CXCL10 were positively correlated with disease activity in vitiligo patients (Wang X. X. et al., 2016). Some cytokines such as KLRG1+ lymphocytes secrete cytotoxic molecules (granzyme B and perforin), inflammatory cytokines (IFN-γ and TNF-α), and inflammatory chemokine receptors (CCR5 and CX3CR1) are involved in gene expression, which are associated with vitiligo (Li et al., 2019). Both *IFITM2* and *CXCL10* are type I IFN (IFN-I) signals, and IFN-I induces the expression of inflammatory mediators (*CXCL10, CCL2, IL-8*, and *BAFF*) (Moschella et al., 2013). Besides, some of the identified DEGs are associated with viral infectious diseases or cancer (De Marco et al., 2018; Litviakov et al., 2018; Zhu et al., 2019; Rchiad et al., 2020). Studies found that the differential expression of human granzyme A, B and K in NK cells and CD8+ T cells in peripheral blood (Grossman et al., 2004; Bratke et al., 2005). *GZMA* expression by virus-specific cytotoxic T lymphocytes was associated with GATA3 binding at the Gzma locus (Nguyen et al., 2016). The findings suggest that vitiligo and these other diseases may have pathogenic pathways or cytokines in common.

We also found that immune cells infiltration in vitiligo was associated with antigen presentation-related gene expression. We observed positive correlations between *DCTN5* and *ERAP2* expression and B cell abundance. Genetic variants of ERAP1 and ERAP2 genes can increase the susceptibility to autoimmune diseases, cancer and infectious diseases (Yao et al., 2019). Chemokine receptors CCR4 and CCR7 play a important role in Treg homing. Their ligands CCL21 and CCL22 could regulate homing of Tregs from regional lymphoid organs to skin (Lim et al., 2006; Sather et al., 2007; Schneider et al., 2007). *NCF2* expression were associated with macrophage abundance (Mitchison et al., 2005; Zhao et al., 2020). The role of antigen presentation-related genes in the pathogenesis of vitiligo have not been reported, which needs further study.

In cell infiltration and immune response-related gene expression, we found that the abundance of endothelial cells in epidermal samples were negatively correlated with CD28 gene expression. The same result was found between CD28 in PBLs and macrophages. The expression of CD28 in PBLs was positively correlated with the abundance of CD4+ T cells. We further analyzed the expression of endothelial cell adhesion molecules in the two skin datasets. No adhesion molecules related gene were found in GSE53146. The abundance of endothelial cells was negatively correlated with CD28 and MPZL1 in GSE75819. The adhesion molecules in endothelial cells included many molecules that mediate the contact and binding between cells or between cells and extracellular matrix. Adhesion molecules in endothelial cells can participate in immune response, inflammation, coagulation, tumor metastasis, wound healing and other processes. They can exist in many parts of the body, such as skin and blood vessels, and have complex mechanisms. It has been reported that inflammatory cytokines IL-1 β and IL-18 can induce Th17 response and endothelial cell damage, aggravate many autoimmune diseases (Yang and Chiang, 2015). Endothelial cells may be related to the pathogenesis of vitiligo, which needs further study.

Our study has several limitations. First, a larger sample size is needed for further analysis. In addition, functional research, including molecular experiments, will be necessary to explore related biological functions. At present, we are collecting damaged skin samples from patients with vitiligo and blood samples from patients and healthy people to verify the immune cell components and associated genes.

In summary, this study confirmed that during the development of vitiligo, the levels of macrophages, B cells and NK cells increase with the activation of T cells. The peripheral blood immune cells play an important role in the pathogenesis of vitiligo. Immune and inflammation related core genes are associated with vitiligo immune cells infiltration. This study provides a new theoretical basis for the clinical diagnosis and treatment of vitiligo.

## Data Availability Statement

The original contributions presented in the study are included in the article/Supplementary Material, further inquiries can be directed to the corresponding author/s.

## Author Contributions

JZ designed the project. SY, WH, MW, and XK contributed on data analysis and prepared the main manuscript. All authors reviewed the manuscript.

## Conflict of Interest

HZ and CC were employed by company ABLife Inc. The remaining authors declare that the research was conducted in the absence of any commercial or financial relationships that could be construed as a potential conflict of interest.

## Footnotes

1 http://www.ncbi.nlm.nih.gov/geo/

2 http://reactome.org

3 https://github.com/icbi-lab/immunedeconv

4 https://github.com/GefellerLab/EPIC

## Supplementary Material

Supplementary Figure 1 Venn diagrams showed the shared probes and DEGs identified from the four transcription profile datasets (GSE53146, GSE75819, GSE80009, and GSE90880). The DEGs were divided into up- and down-regulated groups. Statistically significant DEGs were defined with adjusted P < 0.05 and |log2FC| > 1 as the cutoff criterion. **(A)** Shared probes **(B)** Up-regulated genes **(C)** Down-regulated genes. (https://doi.org/10.6084/m9.figshare.13637828.v1).

Supplementary Figure 2 Analysis of DEGs of skin of vitiligo patients vs healthy controls and non-lesional vs lesional epidermis of vitiligo patients. **(A)** Volcano plot presents DEGs by vitiligo compared with healthy from vitiligo vs healthy dataset (left) and non-lesional vs lesional dataset (right). Red indicates upregulated genes (FC ≥ 1.5 and p value < = 0.05) and blue indicates downregulated genes (FC < = 0.66 and p value < = 0.05). (B–C) Top 10 most enriched GO terms of up **(D)** and down **(E)** genes of non-lesional vs lesional dataset. (https://doi.org/10.6084/m9.figshare.13153874.v7).

Supplementary Figure 3 Analysis of DEGs of peripheral blood of vitiligo patients vs healthy controls. (A–B) Top 10 most enriched KEGG pathways by up **(A)** and down **(B)** regulated genes from vitiligo vs healthy (PBLs) dataset. (C–D) Top 10 most enriched GO terms **(D)** and KEGG terms **(E)** of down-regulated genes from 9 vitiligo vs healthy samples (PBMCs). (https://doi.org/10.6084/m9.figshare.13154144.v3).

Supplementary Figure 4 Cells population analysis in lesional vs non-lesional epidermis of vitiligo patient. **(A)** Principal component analysis (PCA) of samples based on proportion of different cell types. The samples were grouped by disease state and the ellipse for each group is the confidence ellipse. **(B)** Scatter plot for the enrichment of each cell type in lesional vs non-lesional skin dataset. X-axis: log fold change of mean cell fraction of lesional compared to non-lesional skin of vitiligo patients. Y-axis: log p value using Students t-test. **(C)** Box plots showing proportion of each cell type in lesional vs non-lesional skin dataset. (https://doi.org/10.6084/m9.figshare.13154204.v2).

Supplementary Figure 5 Cell type enrichment in peripheral blood of vitiligo patients vs healthy controls. **(A–B)** Scatter plot for the enrichment of each cell type in vitiligo vs healthy (PBMCs) dataset **(A)** and vitiligo vs healthy (PBLs) dataset **(B)**. X-axis: log fold change of mean cell fraction of vitiligo compared to healthy. Y-axis: log p value using Students t-test. (https://doi.org/10.6084/m9.figshare.13154210.v2).

Supplementary Figure 6 Co-expression analysis of cell population and DEGs in peripheral blood and skin of vitiligo patients. **(A)** Top 10 most enriched GO terms (biological process) by cell type co-expressed DEGs in three datasets. **(B)** Gene expression profile involved in macrophage cell ratio correlated immune response and inflammatory response DEGs. (https://doi.org/10.6084/m9.figshare.13154219.v6).
