# Supplementary figures and images for: Comprehensive Analysis of Cell Population Dynamics and Related Core Genes During Vitiligo Development
Source: Front Genet. 2021 Feb 19;12:627092. doi: 10.3389/fgene.2021.627092 (PMC7933673; doi:10.3389/fgene.2021.627092)

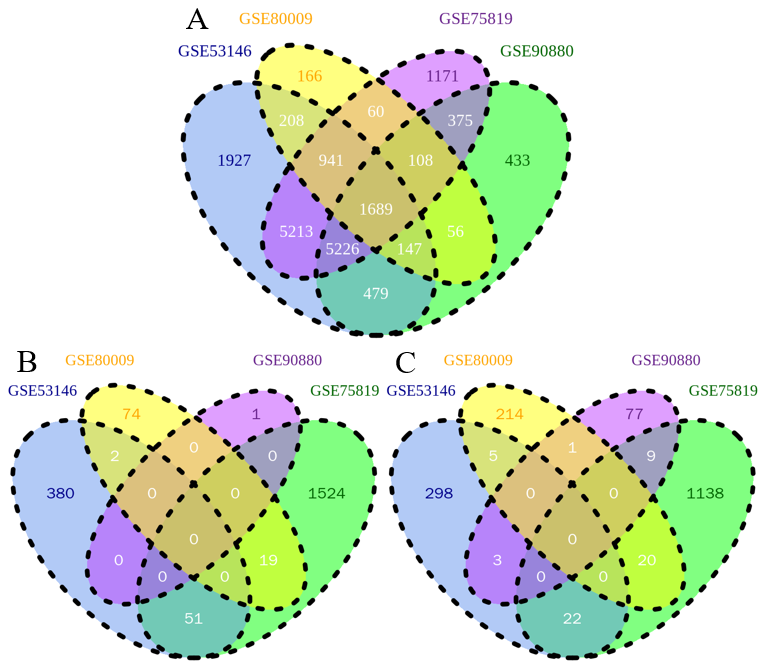

Supplement: Supplementary Figure 1 — Venn diagrams showed the shared probes and DEGs identified from the four transcription profile datasets (GSE53146, GSE75819, GSE80009, and GSE90880). The DEGs were divided into up- and down-regulated groups. Statistically significant DEGs were defined with adjusted P < 0.05 and |log2FC| > 1 as the cutoff criterion. (A) Shared probes (B) Up-regulated genes (C) Down-regulated genes. (https://doi.org/10.6084/m9.figshare.13637828.v1). [file Image_1.TIFF]

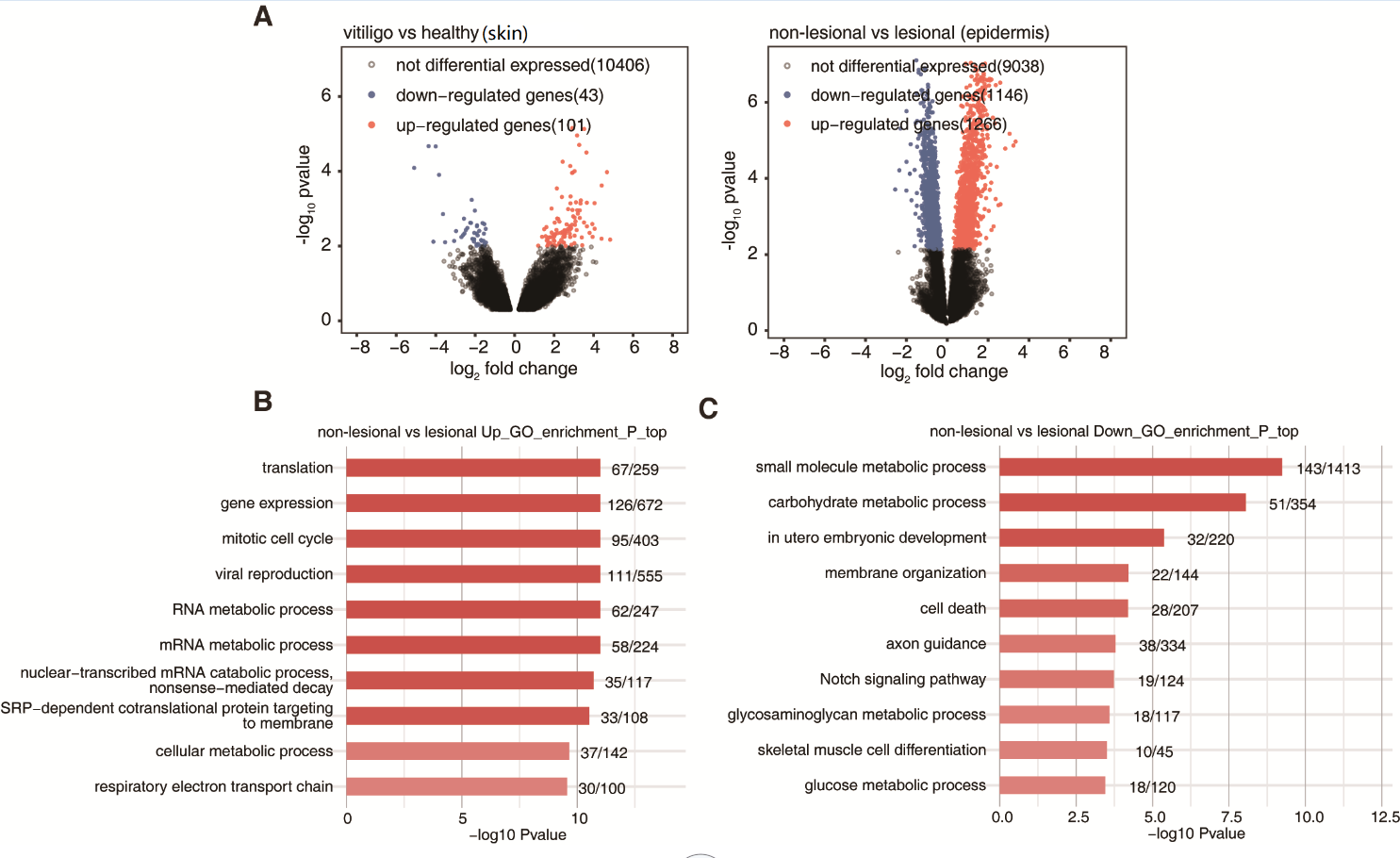

Supplement: Supplementary Figure 2 — Analysis of DEGs of skin of vitiligo patients vs healthy controls and non-lesional vs lesional epidermis of vitiligo patients. (A) Volcano plot presents DEGs by vitiligo compared with healthy from vitiligo vs healthy dataset (left) and non-lesional vs lesional dataset (right). Red indicates upregulated genes (FC ≥ 1.5 and p value < = 0.05) and blue indicates downregulated genes (FC < = 0.66 and p value < = 0.05). (B–C) Top 10 most enriched GO terms of up (D) and down (E) genes of non-lesional vs lesional dataset. (https://doi.org/10.6084/m9.figshare.13153874.v7). [file Image_2.TIFF]

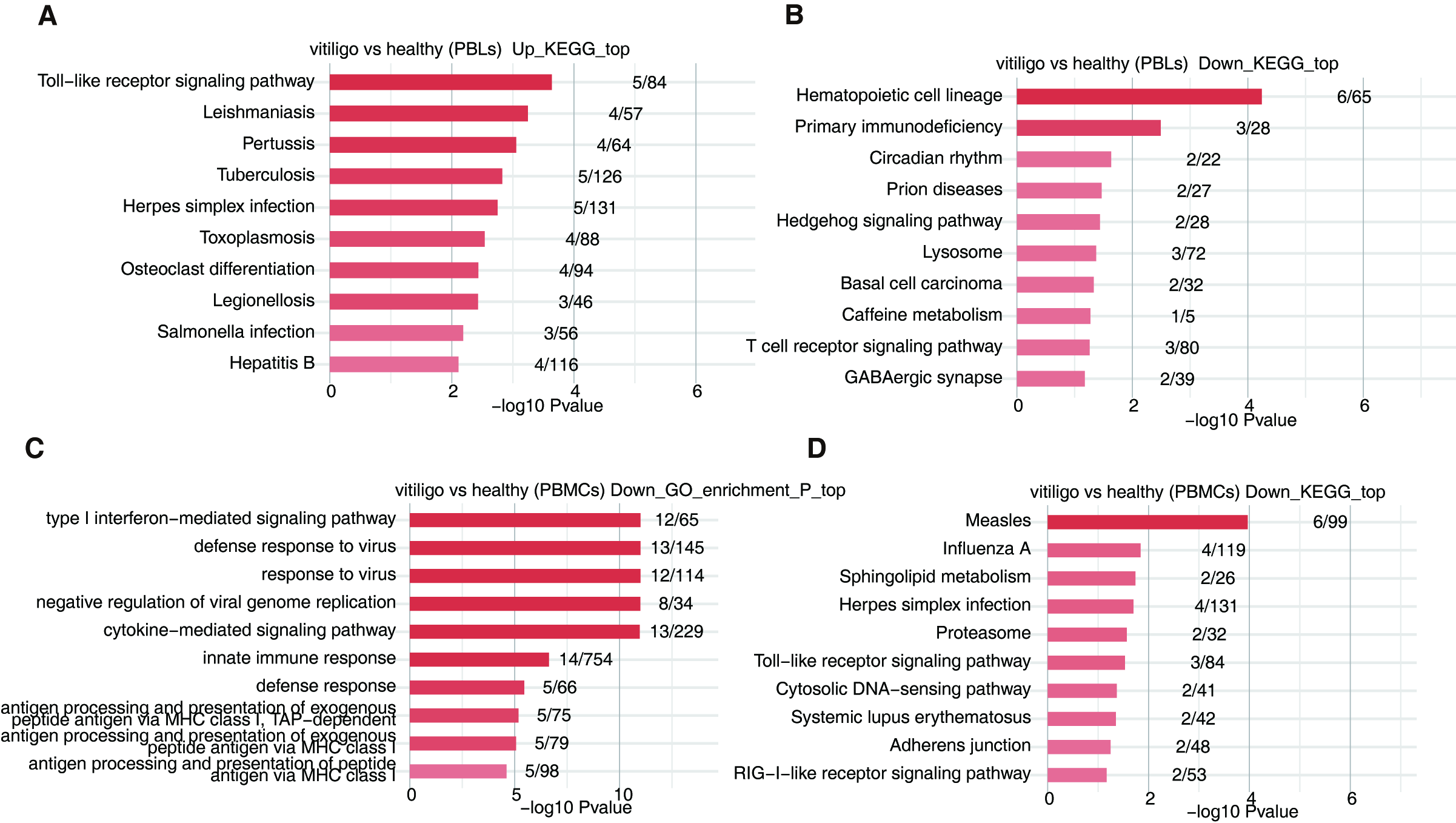

Supplement: Supplementary Figure 3 — Analysis of DEGs of peripheral blood of vitiligo patients vs healthy controls. (A–B) Top 10 most enriched KEGG pathways by up (A) and down (B) regulated genes from vitiligo vs healthy (PBLs) dataset. (C–D) Top 10 most enriched GO terms (D) and KEGG terms (E) of down-regulated genes from 9 vitiligo vs healthy samples (PBMCs). (https://doi.org/10.6084/m9.figshare.13154144.v3). [file Image_3.TIF]

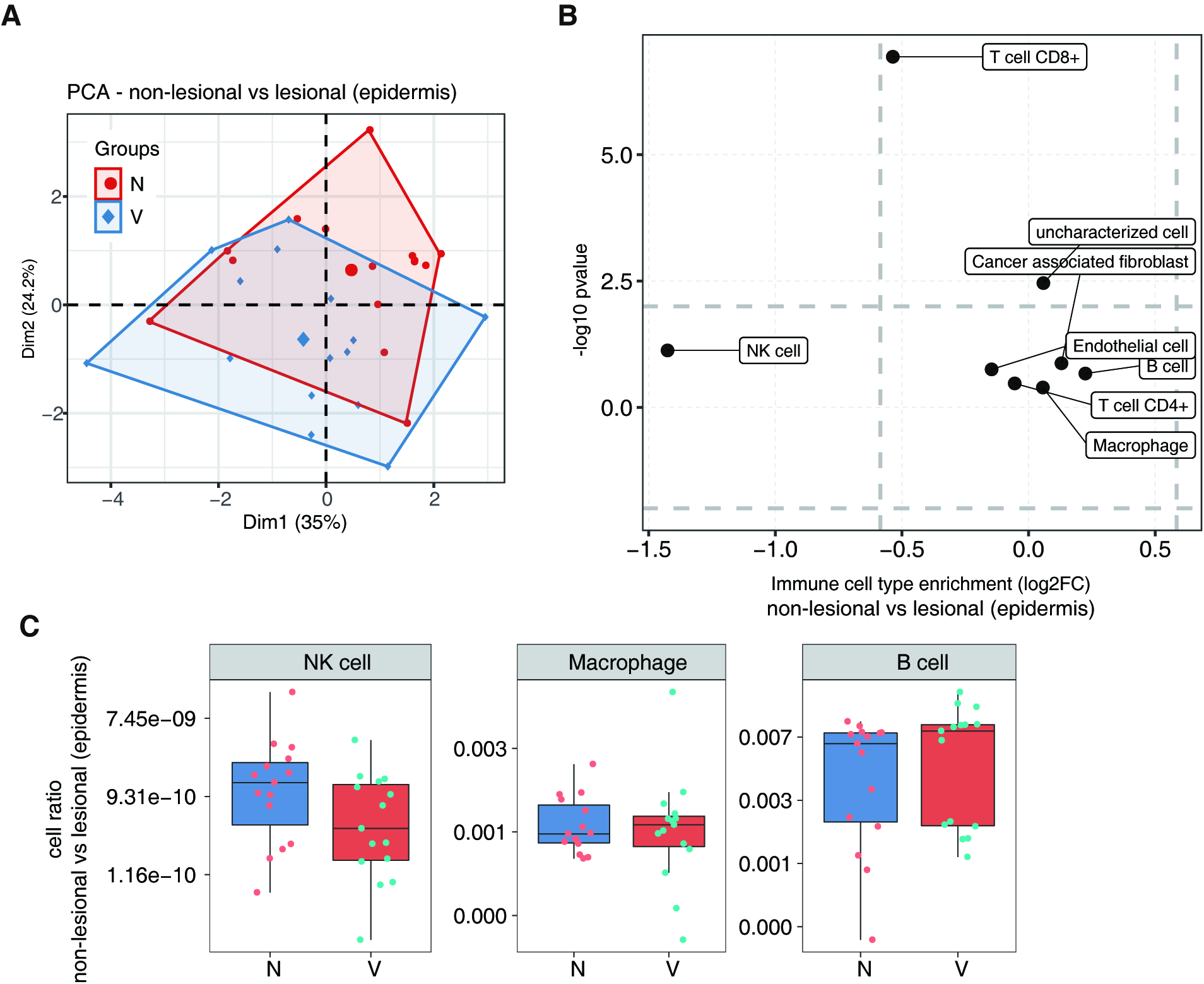

Supplement: Supplementary Figure 4 — Cells population analysis in lesional vs non-lesional epidermis of vitiligo patient. (A) Principal component analysis (PCA) of samples based on proportion of different cell types. The samples were grouped by disease state and the ellipse for each group is the confidence ellipse. (B) Scatter plot for the enrichment of each cell type in lesional vs non-lesional skin dataset. X-axis: log fold change of mean cell fraction of lesional compared to non-lesional skin of vitiligo patients. Y-axis: log p value using Students t-test. (C) Box plots showing proportion of each cell type in lesional vs non-lesional skin dataset. (https://doi.org/10.6084/m9.figshare.13154204.v2). [file Image_4.TIF]

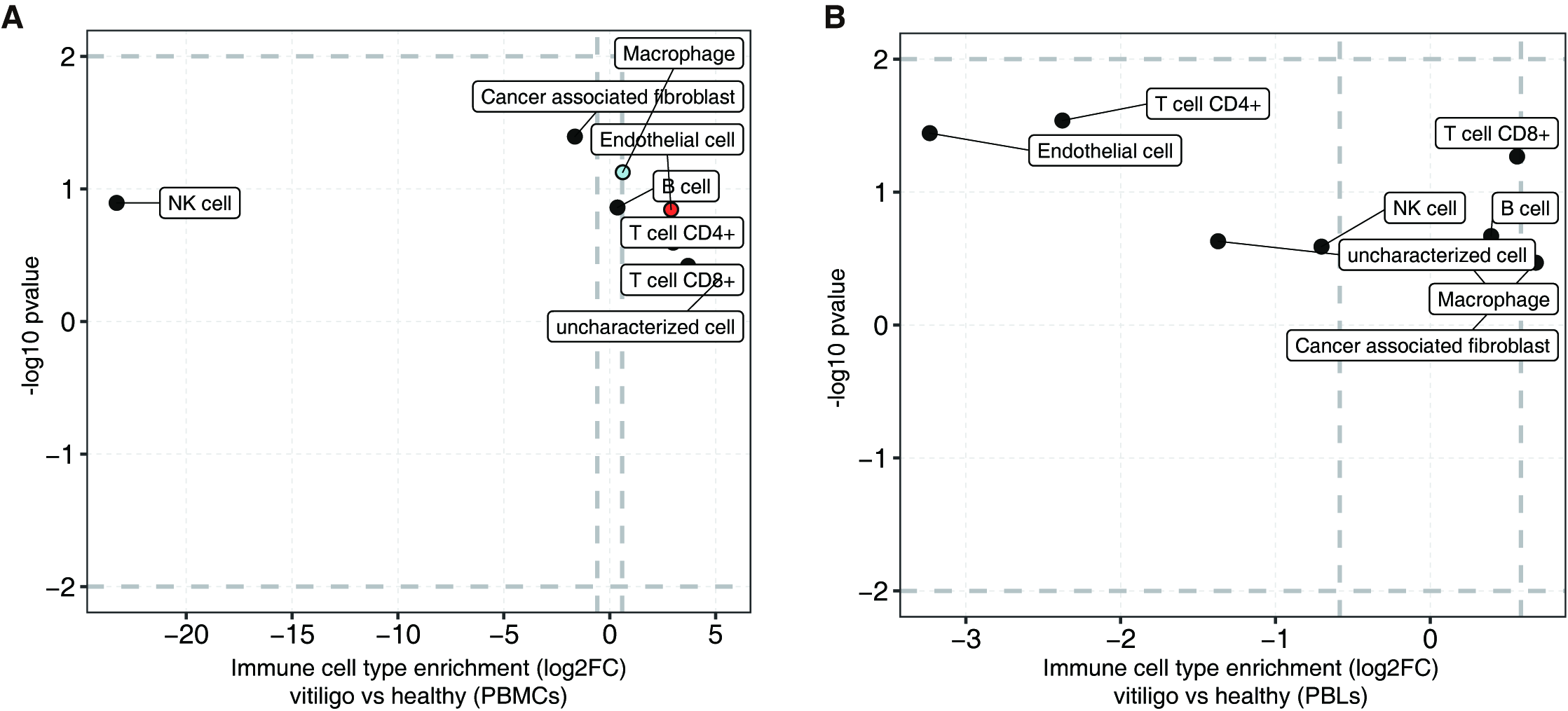

Supplement: Supplementary Figure 5 — Cell type enrichment in peripheral blood of vitiligo patients vs healthy controls. (A–B) Scatter plot for the enrichment of each cell type in vitiligo vs healthy (PBMCs) dataset (A) and vitiligo vs healthy (PBLs) dataset (B). X-axis: log fold change of mean cell fraction of vitiligo compared to healthy. Y-axis: log p value using Students t-test. (https://doi.org/10.6084/m9.figshare.13154210.v2). [file Image_5.TIF]

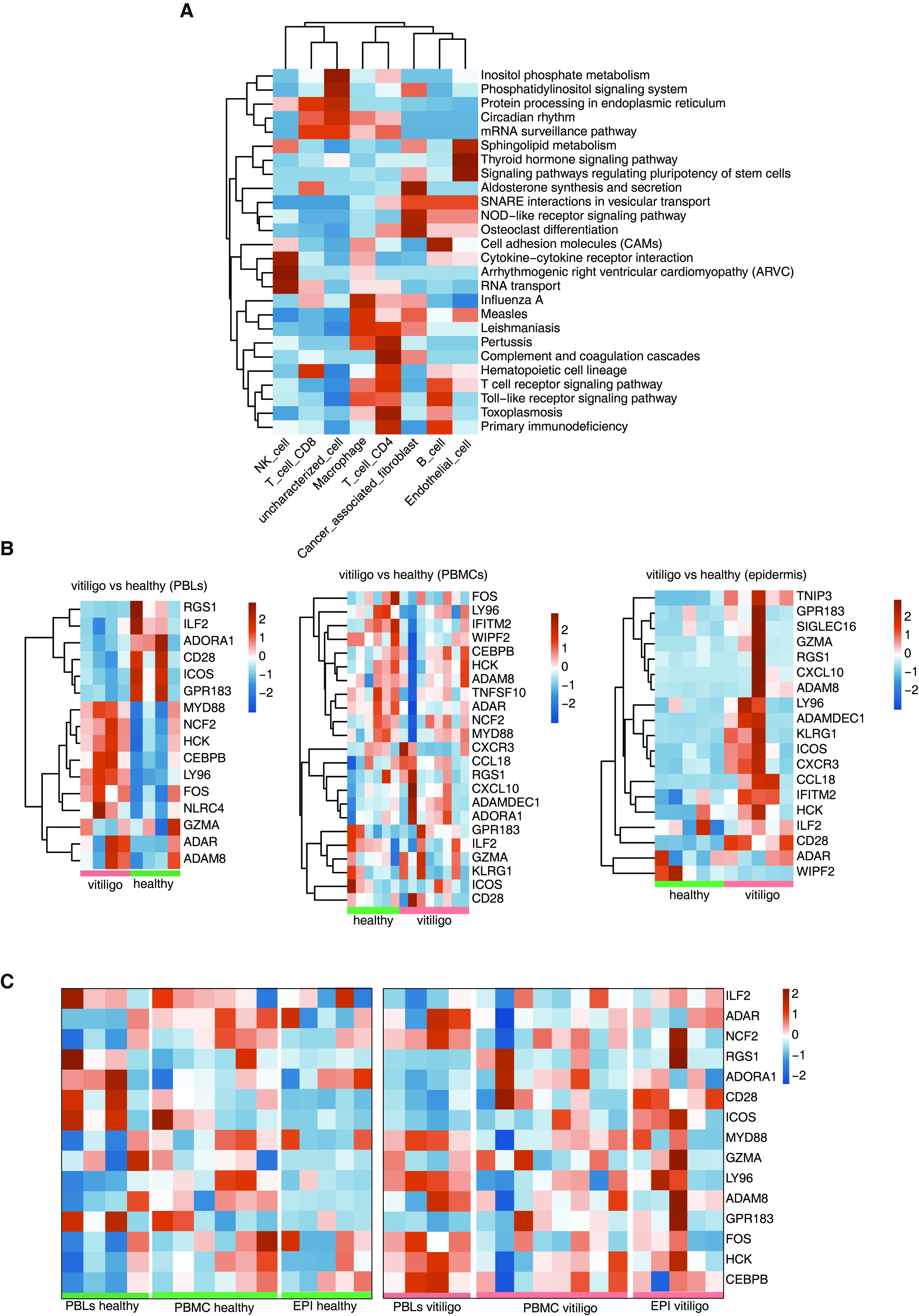

Supplement: Supplementary Figure 6 — Co-expression analysis of cell population and DEGs in peripheral blood and skin of vitiligo patients. (A) Top 10 most enriched GO terms (biological process) by cell type co-expressed DEGs in three datasets. (B) Gene expression profile involved in macrophage cell ratio correlated immune response and inflammatory response DEGs. (https://doi.org/10.6084/m9.figshare.13154219.v6). [file Image_6.TIF]
